# Inflammatory cytokines associated with mild traumatic brain injury and clinical outcomes: a systematic review and meta-analysis

**DOI:** 10.3389/fneur.2023.1123407

**Published:** 2023-05-12

**Authors:** Shazia Malik, Omar Alnaji, Mahnoor Malik, Teresa Gambale, Forough Farrokhyar, Michel P. Rathbone

**Affiliations:** ^1^Neurosciences Graduate Program, McMaster University, Hamilton, ON, Canada; ^2^Faculty of Life Sciences, McMaster University, Hamilton, ON, Canada; ^3^Bachelor of Health Sciences Program, McMaster University, Hamilton, ON, Canada; ^4^Division of Neurology, Department of Medicine, McMaster University, Hamilton, ON, Canada; ^5^Department of Surgery and Department of Health Research Methods, Evidence, and Impact, McMaster University, Hamilton, ON, Canada

**Keywords:** concussion, neuroinflammation, mTBI, cytokines, traumatic brain injury

## Abstract

Mild traumatic brain injuries (mTBIs) trigger a neuroinflammatory response, which leads to perturbations in the levels of inflammatory cytokines, resulting in a distinctive profile. A systematic review and meta-analysis were conducted to synthesize data related to levels of inflammatory cytokines in patients with mTBI. The electronic databases EMBASE, MEDLINE, and PUBMED were searched from January 2014 to December 12, 2021. A total of 5,138 articles were screened using a systematic approach based on the PRISMA and R-AMSTAR guidelines. Of these articles, 174 were selected for full-text review and 26 were included in the final analysis. The results of this study demonstrate that within 24 hours, patients with mTBI have significantly higher levels of Interleukin-6 (IL-6), Interleukin-1 Receptor Antagonist (IL-1RA), and Interferon-γ (IFN-γ) in blood, compared to healthy controls in majority of the included studies. Similarly one week following the injury, patients with mTBI have higher circulatory levels of Monocyte Chemoattractant Protein-1/C-C Motif Chemokine Ligand 2 (MCP-1/CCL2), compared to healthy controls in majority of the included studies. The results of the meta-analysis also confirmed these findings by demonstrating significantly elevated blood levels of IL-6, MCP-1/CCL2, and Interleukin-1 beta (IL-1β) in the mTBI population compared to healthy controls (*p* < 0.0001), particularly in the acute stages (<7 days). Furthermore, it was found that IL-6, Tumor Necrosis Factor-alpha (TNF-α), IL-1RA, IL-10, and MCP-1/CCL2 were associated with poor clinical outcomes following the mTBI. Finally, this research highlights the lack of consensus in the methodology of mTBI studies that measure inflammatory cytokines in the blood, and also provides direction for future mTBI research.

## 1. Introduction

Most traumatic brain injuries are classified as mild traumatic brain injuries (mTBI) or concussions. mTBI induces a variety of symptoms including headaches and other physical, cognitive, and emotional symptoms, commonly referred to as post-concussion symptoms. These symptoms often resolve spontaneously within a few days to months. However, up to 56% of individuals with mTBI either develop prolonged symptoms or do not recover (1–4). Persistent post-concussive symptoms are usually associated with increased healthcare costs, disability, and reduced quality of life (5–9).

Interestingly, post-concussion like symptoms appear to be nonspecific to mTBIs, as they are also seen in individuals who have sustained other bodily injuries (10–14). Following a head injury, a cascade of acute neurochemical, metabolic, and cellular changes are triggered within the brain (15). Neuroinflammation is a secondary consequence of mTBI that appears to be one of many factors associated with post-concussion symptoms (16, 17). It is observed that post-concussion like symptoms are associated with inflammatory cytokines independent of head injuries (18). For example, headache, one of the most common post-concussion symptoms, is associated with elevated levels of Tumor Necrosis Factor-alpha (TNF-α), Interleukin-1 beta (IL-1β) and Interleukin-10 (IL-10) (19, 20). Similarly, depression is associated with elevated C-Reactive Protein (CRP) and Interleukin-6 (IL-6) levels, while anxiety is associated with an increase in CRP, TNF-α, and Interferon-γ (IFN-γ) levels (21–23). The same holds true for chronic subjective dizziness which is associated with elevated TNF-α and IFN-γ levels (24). Furthermore, IL-1β is associated with benign paroxysmal positional vertigo (BPPV) (25). This indicates that inflammation is associated with various post-concussion like symptoms, irrespective of the triggering cause.

To establish an association between the inflammatory cytokines and mTBI-related symptoms, the best approach one can take is to measure cytokine levels intracranially. Since mTBI is a minor injury with no signs of obvious trauma on routine imaging, it is not feasible to undertake a lumbar puncture to measure intracranial cytokine levels. To overcome this issue, many studies attempting to study inflammation in mTBI measure cytokine levels peripherally in blood. However, as mentioned earlier, inflammation is not exclusive to mTBI, so while measuring cytokine levels peripherally is convenient, it can present issues in differentiating the source of inflammation. Hence, it is important to characterize the inflammatory cytokine profile that is unique to patients with mTBI, both in blood and CSF.

A systematic review and meta-analysis were conducted to examine and analyze the evidence presented from clinical studies linking mTBI with various inflammatory cytokines, both in blood and CSF. Our primary aim is to compare the inflammatory cytokine levels between populations with mTBI and healthy control (HC) groups. The secondary aim is to compare the inflammatory cytokine levels between the population with mTBI and trauma control (TC) groups. Finally, the last aim is to explore the associations between the post-mTBI inflammatory cytokine levels and clinical outcomes and prognosis. This research would help us identify the inflammatory cytokine profile exclusive to mTBI, that sets it apart from healthy controls as well as trauma controls. In addition, this research would help us identify the inflammatory cytokines that have the most potential to be used as prognostic mTBI biomarkers.

## 2. Methodology

### 2.1. Search strategy

A systematic screening approach in fulfillment of the Preferred Reporting Items for Systematic Reviews and Meta-analyses (PRISMA) and the Revised Assessment of Multiple Systematic Reviews (R-AMSTAR) guidelines was implemented (26). Potentially eligible studies were identified by systematically searching the databases PUBMED, EMBASE and MEDLINE. The searches were limited to literature published from January 2014 to December 12, 2021. Studies published prior to January 2014 were discussed in our previous study (18). The search strategy was developed using combinations of the following MeSH terms: (“mild traumatic brain injur*” OR “concussion”) AND (“neuroinflammat*” OR “cytokine*”). A secondary manual search, using Google Scholar, was also conducted to ensure that all relevant articles were captured.

### 2.2. Study screening

Citations were uploaded into Covidence for title, abstract, and full-text screening as well as duplicate removal. Two independent reviewers conducted the study screening in duplicate, from title to full-text screening stages (SM and OA). Disagreements regarding article inclusion were settled by mutual consensus after discussing the disputed articles together. Any further discrepancies were discussed with other team members and eventually resolved by the principal investigator (TG and MR).

### 2.3. Selection criteria

Only studies providing information on inflammatory cytokines in the CSF, blood, plasma, or serum of the patients with mTBI were considered for review. The research question and inclusion/exclusion criteria were established *a priori*. Inclusion criteria were defined as: (1) concussions or mild traumatic brain injuries, (2) neuroinflammation, (3) inflammatory cytokines, and (4) articles published in English. Exclusion criteria were defined as: (1) complicated mild and more severe forms of traumatic brain injuries with Glasgow Coma Scale (GCS) < 13, (2) no blood or CSF cytokines, (3) no comparison healthy or trauma controls or baseline (pre-mTBI) groups, (4) review articles, abstracts or letter to editors, (5) cadaver/non-human studies, and (6) articles that included TBIs but did not make distinctions between the various types of TBIs. The reference lists of related studies were also searched for additional reports.

### 2.4. Data extraction

Two independent reviewers (SM and MM) abstracted the relevant data from the included articles on an Excel sheet. The characteristics extracted from each study included the author, publication year, study design, sample size, mTBI setting, mTBI diagnostic criteria, control type, and patient demographics (e.g., age, sex, etc.). Details about the number of previous mTBIs, time since last mTBI, and GCS data were also recorded. Furthermore, data regarding methods of cytokine measurement, type of biospecimen analyzed, the time interval between injury and cytokine measurement, cytokine levels (both mTBI and control groups), along with any relevant *p*-values, were recorded. In addition, any acute or chronic functional outcomes associated with a particular cytokine were noted. This included the presence of persistent symptoms, reduced or lack of return to normal activities (work, school, and sports), abnormal neurocognitive function, and Glasgow Outcome Scores (GOSE).

To conduct the meta-analysis, the mean cytokine concentrations, and their standard deviations (SD) for case and control groups were extracted at each follow-up visit. The timing of cytokine concentration measurement varied from <24 h to >1 month. Other descriptive statistics such as medians and measures of variance (e.g., 95% confidence intervals [CI] and range) were also extracted. Efforts were made to contact the authors of studies that presented their data as either medians, or in a log-transformed format only, to ensure that all possible mean values were available to conduct a thorough meta-analysis. Data from studies that had overlapping populations was extracted but a distinction was made in the analysis.

### 2.5. Reporting quality

Risk of bias and study quality were evaluated using the Newcastle-Ottawa Quality Assessment Scale (NOS) (27). A score of 0 or 1 was given for each category/criterion on the NOS scale, where the maximum possible score of 8/8 could be achieved (maximum score of 1 for each category). The total scores were categorized according to the methodological quality of each study. Potential confounding factors (including type of biospecimen, assay type, time since mTBI, type of mTBI population and control for confounding inflammatory variables etc.) were also considered for a more detailed bias and quality analysis of studies.

### 2.6. Data analysis

Review Manager (RevMan) Version 5.4 (The Cochrane Collaboration, 2020) was used for the meta-analysis. Meta-analyses were conducted whenever the mean values of an inflammatory cytokine were available in at least three or more studies, with a minimum of 30 participants in each study. For the studies that did not report SD, it was calculated from SEM (Standard error of mean) and CI 95% (Confidence Interval 95%) using the following formulas:


$$\mathrm{SEM}=\frac{\mathrm{Upper limit of}\;\mathrm{CI}\;95\%-\mathrm{Lower limit of}\;\mathrm{CI}\;95\%}{3.92}$$

$$\mathrm{SD}=\mathrm{SEM}\times \sqrt{n\;}\left(\mathrm{number of participants}\right)$$

Due to a limited number of studies that qualified for the meta-analysis, only four analyses were conducted comparing mean TNF-α, IL-6, IL-1β, and MCP-1/CCL2 levels in serum/plasma/blood between patients with mTBI and healthy control groups. A high level of heterogeneity was expected due to the utilization of different assay methods (i.e., Multiplex and ELISA), time of cytokine measurement, control for inflammatory variables, and different blood fractions and dilutions used. To account for this heterogeneity, a random effects model and inverse variance approach were utilized to estimate the pooled standardized (Std.) mean differences, their corresponding 95% confidence interval, and *p*-values. The Std. mean difference is used when the included studies measure the same outcome in different ways. It standardizes the differences in the measurement of the same outcome before pooling the means. It does not however remove the heterogeneity among the study population. Random effects models are preferable if significant heterogeneity is expected as this model accounts for both within-study variability and between-study variability. Heterogeneity was tested using Cochrane’s Q test with the *p*-value set at 0.1 for significance and quantified using the *I*^2^ statistic (*I*^2^ > 40% as low, 40–60% as moderate, and > 60% as substantial heterogeneity). The sources of heterogeneity were evaluated and the risk of bias across studies, publication bias, and selective reporting were assessed. Sensitivity analyses were conducted by excluding the studies in which mean and standard deviation were estimated (from reported standard deviations and range values) to assess the consistency of the estimated mean differences. If multiple studies conducted by the same group had overlapping populations, only the most recent study with the largest mTBI population size was included in the meta-analysis. If cytokines were measured at different time points, the time-point with the largest population size was used for the total cytokine analysis. For acute and chronic cytokine analysis, the most time-appropriate data consistent with the timings of the other studies was used.

## 3. Results

### 3.1. Study characteristics

**Figure 1:**
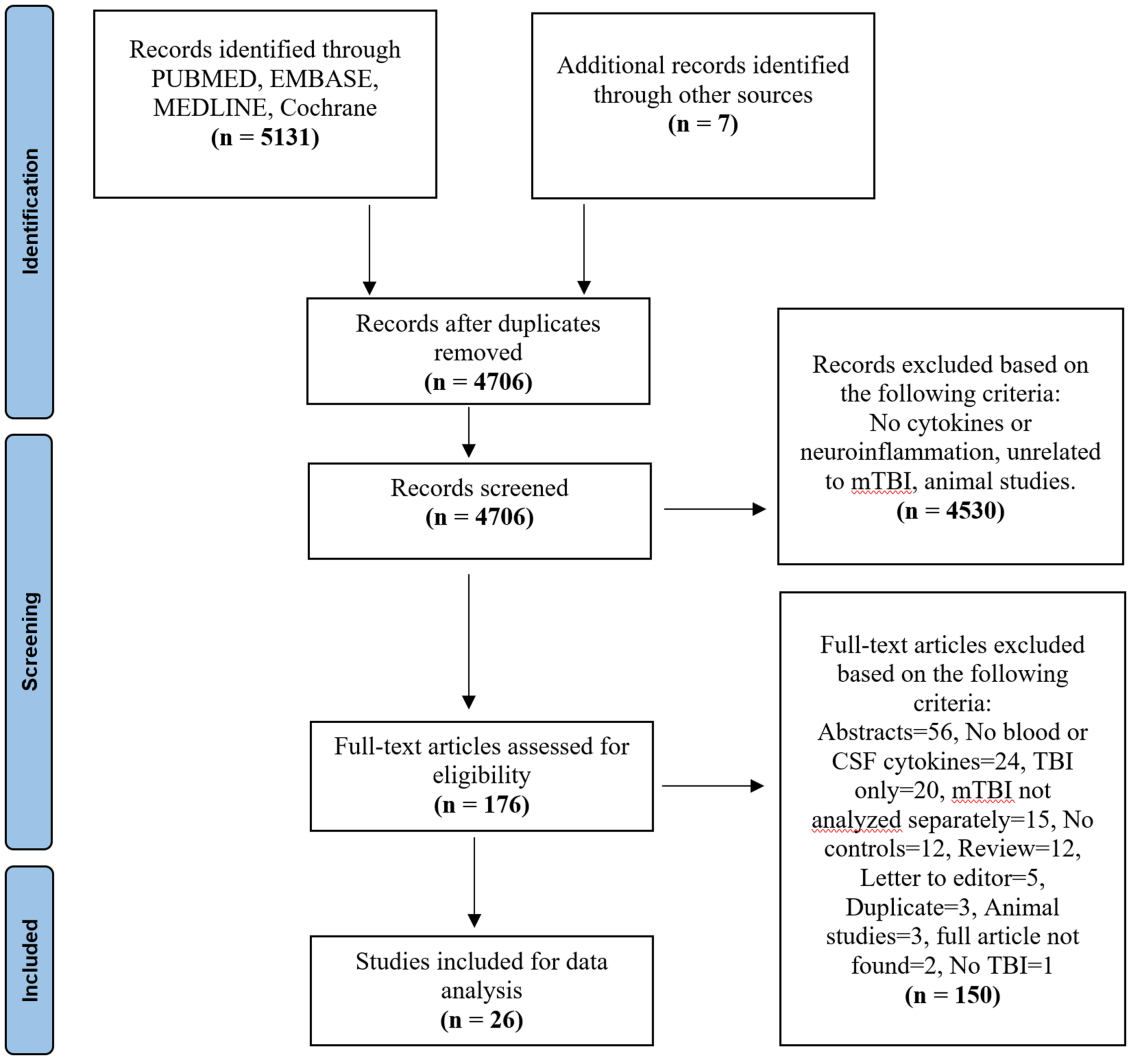
PRISMA flow chart.

A total of 5,138 studies were yielded across the three databases Embase, 139 from Medline, 662 articles from PubMed, and 7 from other sources. After removing duplicates, a systematic screening process was conducted as shown in Figure 1, yielding a total of 26 articles that met the selection criteria (Figure 1). Out of the 26 studies, 25 compared blood cytokine levels between patients with mTBI and healthy controls and 3 studies compared the levels between patients with mTBI and trauma controls (some studies had both control types). Only one study compared cytokine level differences in the CSF. This CSF data was not included in the qualitative or quantitative analysis, but the results are available in Table 1. The characteristics of the 26 included studies are described in Table 1.

**Table 1:** Study characteristics.

Author year	Population	Biomarkers tested	Biomarker assessment	Specimen used	Sig. data	Time (acute/chronic)	mTBI Dx/setting	Variable control	Prognosis/outcome
Shan et al. (47)	mTBI = 55 TC = 17 HC = 44	TNF-α, IL-1β, CXCL1, CXCL8, and CCL2	ELISA (R&D Systems, United States)	Plasma	None of the biomarkers selected have a sig. difference between the groups.	Acute mTBI (1–8) hours mTBI (9–24) hours OI < 24 h	Zurich 2012/general trauma	Yes	NR
Meier et al. (28)	mTBI = 106 HC = 134	IL-6, IL-1RA, and CRP	Multiplex (Meso Scale Diagnostics, United States)	Serum	IL-6, IL-1RA, and CRP are sig. elevated at <6 h in mTBI groups compared to healthy (*p* = 0.001) IL-1RA is sig. elevated at 24–48 h in mTBI groups compared to healthy (*p* < 0.05).	Acute Pre-injury baseline Within 6 h 24–48 h	CDC/SRC	Partial	Elevated IL-1RA (*p* = 0.03) and IL-6 (*p* = 0.08) ass. with symptom duration.
Nitta et al. (29)	mTBI = 40 HC = 43	IL-6, IL-1β, IL-1RA, IL-10, TNF-α, CRP, and IFN-γ	Multiplex (Meso Scale Diagnostics, United States)	Serum	IL-1RA and IL-6 levels at 6 h visit are sig. higher in athletes with mTBI (*p* < 0.001).	Acute Baseline (6 h) 24–48 h Days 8, 15, and 45	CDC/SRC	Partial	IL-6 levels at 6 h ass. with the duration of symptoms (*p* = 0.031).
Feng et al. (48)	mTBI = 16 HC = 11	TNF-α	ELISA (Biotech Co., China)	Plasma CSF	Plasma TNF-α is sig. higher in mTBI compared to controls (*p* = 0.009) Day 3 plasma TNF-α is sig. higher than day 1, 5, and 7 (*p* < 0.05) CSF TNF-α levels higher (non-sig) in mTBI patients at Day 3	Acute Days 1, 3, 5, and 7	GCS/general trauma	Yes	NR
Goetzl et al. (45)	mTBI = 32 HC = 21	IL-6	ELISA (R&D Systems, United States)	Plasma NDE levels	IL-6 sig. Increased in both acute (*p* < 0.0001) and chronic (*p* < 0.1) mTBI compared to controls.	Acute: 7 days Chronic: 3–12 months	NCAA/SRC	No	NR
Battista et al. (40)	mTBI = 41 HC = 55	IL-6	Ella™ (Protein Simple, Biotechne, United States).	Plasma	No sig. results (differences).	Acute <7 days	Berlin 2016/SRC	No	No sig. correlation between IL-6 and either symptom burden or days to medical clearance (*p* > 0.05).
Tylicka et al. (51)	mTBI = 29 HC = 13	IL-8, IL-11	ELISA (R&D Systems, United Kingdom).	Plasma	IL-8 sig. higher in mTBI (*p* = 0.033)	Acute 2–6 h	GCS/general trauma	Yes	NR
Rusiecki et al. (41)	mTBI = 90 HC = 50	IL-1α, IL1β, IL4, IL-6, IL8, IL-10, TNFα, MCP-1, IL-13, IL-17, TNF-β	Multiplex (Ray Biotech, United States)	Serum	Controls’ cytokine levels are greater than cases’ for IL-6 (*p* = 0.02), IL-8 (*p* = 0.01) and IL-1β (*p* = 0.05)	Chronic 281.8 days (mean)	DoD-VA criteria/military	Partial	Decreased IL-8 levels ass. with PTSD (*p* = 0.01)
Begum et al. (52)	mTBI = 23 HC = 12	92 cytokines	Multiplex (Olink Biosciences, Sweden)	Serum	IL-7 levels sig. Increased in mTBI (*p* < 0.05) MCP-1 was sig. reduced in mTBI at >1 week (*p* = 0.03) CXCL1 was sig. increased in mTBI at <1 week (*p* = 0.02)	Acute: 2–5 days Chronic: 15–75 days	ACRM/SRC	Partial	Reduced MCP-1 levels relate to an increase in the number (*r* = 0.455, *p* = 0.013) and severity of symptoms (*r* = −0.378, *p* = 0.043).
Vedantam et al. (53)	mTBI = 53 TC = 12	IL-1β, IL-2, IL-4, IL-6, IL-10, IL12p70, IL-17a, IFNγ, TNFα	Luminex Magpix (Luminex, United States)	Plasma	Sig. elevated IL-2 (*p* = 0.014) and IL-6 (*p* = 0.01) levels in mTBI within 24 h post-injury. Sig. elevation in IL-6 (*p* = 0.044) at 6 months post-injury in mTBI.	Acute: < 24 h Chronic: 6 months	ACRM/general trauma	No	At 24 h, elevated IL-2 (*p* = 0.001) and lower IL-6 (*p* = 0.035) and IL-17a levels (*p* = 0.007) ass. with severe PCS at 1 week (*p* = 0.001). At 6 months, elevated IL-10 ass. with depression (*p* = 0.004) and PTSD (*p* = 0.001).
O’Brien et al. (50)	mTBI = 58 HC = 47	IL-1β and IL-18	Simoa (Quanterix, MA)	Serum	No sig. results (differences).	Acute: Baseline, 2, 6, and 13 days	NR/SRC	Partial	NR
Sun et al. (33)	mTBI = 95 HC = 54	CCL2, IL-1β, IL-4, IL-6, IL-8, IL-10, IL-12, IFN-γ, TNF-α	Multiplex (Luminex, United States)	Serum	CCL2, IL-1β, and IL-6 levels (acute) higher in mTBI at all time points compared to HC (*p* < 0.001), except IL-1β at 3 months time-point.	Acute and Chronic Cohort 1: within 7 days post injury, 1 month, 3 months Cohort 2: within 7 days post injury	WHO/general trauma	Yes	Elevated CCL2 level ass. with more severe PCS (*p* < 0.001) and predicted information processing speed at 3 months (*p* = 0.009). IL-1β is negatively ass. with working memory in acute phase (*p* < 0.001) and positively in chronic phase (*p* = 0.015).
Battista et al. (39)	mTBI = 42 TC =30 HC = 102	IFN-γ, IL-8, TNF-α, MCP-1, MCP-4, MCP-1β, MIP-1α.	Multiplex (Meso Scale Diagnostics, United States)	Plasma supernatant	Patients with mTBI have higher levels of MCP-4 (*p* < 0.001) and MIP-1β (*p* = 0.001) compared to HC.	Acute 2–7 days	Berlin 2016/SRC	No	MCP-1 (*p* = 0.007) and MCP-4 (*p* < 0.001) positively correlate with days to recovery in mTBI patients.
Guedes et al. (35)	mTBI = 150 HC = 45	TNF-α, IL-6, IL-10,	Simoa (Quanterix, Lexington, MA)	Plasma	No sig. differences in the plasma or exosomal concentrations of any biomarker.	Chronic 6.83–9.53 years (median)	DoD–VA/military	No	PCS severity correlate with plasma TNF-α (*r* = −0.2328, *p* = 0.02). PTSD correlated weakly with plasma TNF-α (*r* = −0.2267, *p* = 0.0255). A marginally sig. correlation between PTSD and exosomal IL-6 (*r* = 0.1893, *p* = 0.08).
Gill et al. (34)	mTBI = 42 HC = 22	TNFα, IL-6, IL-10	Simoa (Quanterix, Lexington, MA)	NDEs from blood	mTBI has elevated concentrations of IL-10 (*p* < 0.05).	Chronic >3 months	WARCAT/military	Yes	Exosomal IL-10 levels are related to PTSD symptoms (*B* = 0.8, *t* = 2.60, *p* < 0.01). IL-10 regression model (*p* < 0.01) shows PTSD sig. related (*p* < 0.01) and depression (*p* = 0.063) and PCS severity do not relate to exosomal IL-10 (*p* = 0.26).
Thompson et al. (42)	mTBI = 171 HC = 122	IL-1β, IL-2, IL-4, IL-5, IL-6, IL-7, IL-8, IL-10, IL-12, IFN-γ and TNF-α	Multiplex (Bio-Rad, United States)	Plasma	Within 24 h of injury, concentrations of IL-1β, IL-2, IL-4, IL-5, IL-6, IL-7, IL-8, IL-10, IL-12, IFN-γ, and TNF-α were sig. elevated in mTBI. At 1 month, TNF-α, Il-7 and IL-8 levels were sig. elevated in mTBI. At 6 months, TNF-α, IL-7, IL-8 and IL-12 were sig. elevated in mTBI. These comparisons are for ages 21–54.	Acute: <24 h Chronic: 1 and 6 months.	CDC/general trauma	Yes	NR
Edwards et al. (37)	mTBI = 45 HC = 49	IL-6, IL-10, and TNF-α	Simoa (Quanterix, Lexington, MA)	Serum	At <8 h IL-6 levels in mTBI are greater than HC (*p* < 0.001). No sig. differences at the second time point.	Acute < 8 h and 24 h later	DoD–VA/military	Yes	NR
Kanefsky et al. (36)	mTBI = 61 HC = 82	TNF-α, IL-6 and IL-10	Simoa (Quanterix, Lexington, MA)	Plasma	IL-6 elevated in the mTBI w LOC group compared to both the mTBI w/out LOC and control groups (*p* < 0.001 for both comparisons).	NR	WARCAT/military	Yes	Increased TNF-α in mTBI ass. with severe PTSD (*r* = 0.36, *p* = 0.005). mTBIs with LOC are ass. with elevated IL-6 levels and pain, compared to mTBI without LOC and HC.
Brahmajothi and Abou-Donia (46)	mTBI = 5 HC = 5	TNF-α, IL-6,	ELISA (R&D Systems, United States)	Plasma	TNF-α and IL6 sig. elevated in chronic stages, but not acute (*p* < 0.0001).	Acute: baseline Chronic: 1–2 yrs.	NR/military	No	NR
Powell et al. (43)	mTBI = 55 HC = 49	IL-6	ELISA (ALPCO Diagnostics, United States)	Venous blood	No sig. results (differences).	Chronic 1+ yrs.	Self reported/military	No	NR
Bai et al. (32)	mTBI = 112 HC = 72	IL-6, CCL2, IL-1B	Multiplex (Luminex, United States)	Serum	IL-1β, IL-6, and CCL2 acutely elevated in mTBI relative to HC (all for *p* < 0.001).	Acute <7 days	WHO/general trauma	Partial	NR
Brett et al. (30)	mTBI = 73 HC = 128	IL-6, IL-1RA, and CRP	Multiplex (Meso Scale Diagnostics, United States)	Serum	No sig. results (differences).	NR	DoD-VA/SRC	Partial	Sig. interaction between prior mTBI and IL-1RA levels on the ImPACT memory composite, *p* = 0.044. At low levels of IL-1RA, athletes with multiple mTBI had worse memory performance than those without prior mTBI (*p* = 0.014). Higher IL-1RA levels sig. ass. with more symptoms (elevated BSI-GSI scores, *p* = 0.046) and worse memory (*p* = 0.017).
Chaban et al. (49)	mTBI = 207 HC = 207	IFN-γ, IL-8, IL-9, TNF-α, IL-1RA, MCP-1	Multi-plex (Bio-Rad, Unitd States)	Plasma	IFN-γ, IL-8, IL-17A, IL-9, MCP-1 and TNF-α were sig. higher in mTBI than HC at all time points.	Acute: <72 h and 2 weeks. Chronic: 3 and 12 months.	WHO/general trauma	Yes	NR
Battista et al. (38)	mTBI = 16 HC = 27	MCP-1, MCP-4	Multiplex (Meso Scale Diagnostics, United States)	Venous blood	MCP-1 and MCP-4 were elevated in acute mTBI.	Acute <7 days	NR/SRC	Partial	NR
Ryan et al. (44)	mTBI = 104 HC = 98	IL-2, IL-4, IL-6, IL-8, IL-10, IL-17A, IFN-γ TNF-α	Multiplex (Meso Scale Diagnostics, United States)	Peripheral blood plasma	IL-6 and IL-1RA sig. elevated in mTBI (*p* < 0.005). IL-8, IL-10, IL-17A, TNF-α sig. reduced (*p*-value <0.0001) in mTBI.	Acute: Less than 24 h	GCS 14–15/SRC	Yes	NR
Meier et al. (31)	mTBI = 23 HC = 47	IL-6, IL-10, IL-1β, IL-1RA and TNF-α	Multiplex (Meso Scale Diagnostics, United States)	Serum	Serum IL-6 and IL-1RA levels sig. elevated in mTBI relative to baseline levels (*p* < 0.05).	Acute Pre-injury baseline Within 6 h	CDC/SRC	Partial	IL-6 levels at 6 h are sig. positively ass. with symptom duration (*p* = 0.042) but not Il-1RA levels (*p* = 0.12).

A total of 3,248 participants were included across all studies, where 1,746 of these patients had at least one mTBI. There were 1,502 controls, 1,431 of which were healthy controls, and 71 were trauma controls. The mean sample size of patients with at least one mTBI was 67.15 ± 50.11 whereas, the mean sample size for controls was 57.24 ± 39.02. The mean age of patients with at least one mTBI was 27.37 years, and the mean age of control patients was 27.14 years. 74.5% of mTBI patients were male. Sport-related injuries were the most common source of mTBI in the majority of included studies (42%), followed by general trauma (31%), and military-related injuries (27%). The diagnostic criteria used for mTBI diagnosis was heterogenous.

### 3.2. Study quality

Most studies in this review have a level of evidence of IV (*N* = 14; 53.8%). There was substantial agreement between the two reviewers at the title/abstract screening stage (*κ* = 0.80 [95%CI, 0.70–0.90]) and the full-text screening stage (*κ* = 0.79 [95%CI, 0.60–0.90]). The mean NOS score for the included studies was 6.38 ± 1.19, which indicates a fair quality of evidence for non-randomized studies. The areas of best performance based on the NOS checklist were the case definition (*N* = 25; 96%) and the definition of controls (*N* = 25; 96%). The area of worst performance was the non-response rate (the number of patients that were lost to follow-up), which was not provided in any of the included studies.

### 3.3. mTBI vs. healthy controls: qualitative review of blood inflammatory cytokines

**Figure 2:**
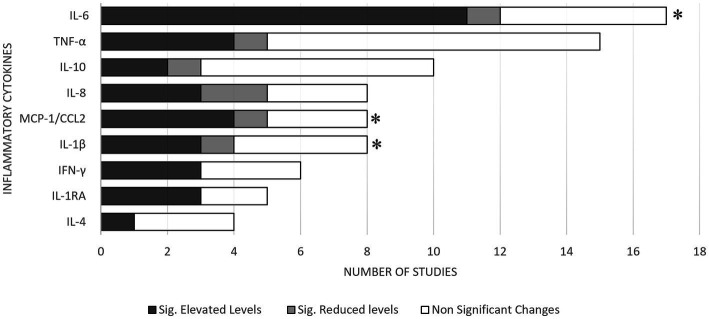
Summary of results for mTBI vs. healthy controls. * Meta-analysis demonstrated acutely elevated levels of the respective cytokines in patients with mTBI.

The most common blood inflammatory cytokines assessed in the included studies were IL-6, TNF-α, IL-10, IL-1β, Interleukin-8 (IL-8), IFN-γ, Interleukin-1 Receptor Antagonist (IL-1RA), Interleukin 4 (IL-4), and MCP-1/CCL2 (Figure 2). It should be noted that MCP-1 is also referred to as CCL-2 and both these terms are used interchangeably. Most of the included studies extracted peripheral inflammatory cytokine specimens from plasma (46%, *n* = 12). The remaining studies extracted inflammatory cytokines from either serum (38%, *n* = 10) or whole blood (15%, *n* = 4). About 42% of the studies (*n* = 11), assessed cytokine levels within 24 hrs of injury. However, most studies (53.8%, *n* = 14) assessed cytokine levels 30 days or later following a mTBI. The systematic review found elevated levels of IL-6 (time points: <24 h, 1–7 days and ≥ 30 days), TNF-α (≥30 days), IL-1β (1–7 days), IL-8 (time points: <24 h and ≥ 30 days), IFN-γ (<24 h), IL-1RA (<24 h), and MCP-1/CCL2 (time points: 1–7 days and ≥ 30 days) in patients with mTBI, compared with healthy controls, where any significant findings were replicated in at least two studies. The evidence was particularly strong for IL-6, IFN-γ, IL-1RA levels (at <24 h), and MCP-1/CCL2 (between 1 and 7 days), where ≥60% of the studies found significant elevated levels in patients with mTBI compared to healthy controls at these time points.

There are four groups of research articles that had subject overlap, where the participant pools were utilized multiple times by researchers in the same group. These groups are labeled as Groups A, B, C, and D. Group A published four of the included studies (28–31), Group B published two studies (32, 33), Group C published four studies (34–37), and finally Group D published three articles (38–40). For this review, clear distinctions were made if two or more studies had overlapping populations at a certain time point. This is to avoid any potential impact on the statistical analysis and the results of this review, caused by falsely giving undue weight to a certain study population.

#### 3.3.1. IL-6

Blood IL-6 levels were assessed in 65% of the included studies (*n* = 17/26) (28–37, 40–46) (Supplementary Figure S1). Out of these, most studies (65%, *n* = 11/17) showed significantly elevated IL-6 levels in patients with mTBI at a minimum of one time-point compared to healthy controls (28, 29, 31–33, 36, 37, 42, 44–46). On the other hand, one study showed significantly reduced IL-6 levels in the mTBI population compared to healthy controls (5.8%, *n* = 1/17) (41). The remaining studies showed no significant differences between the two populations at any time point (29.4%, *n* = 5/17) (30, 34, 35, 40, 43). It should be noted that there was a subject overlap to some extent between the four studies conducted by Group A (28–31), two by Group B (32, 33) and three by Group C (34–36).

Within 24 h, six out of seven studies measuring IL-6 showed elevated levels in the mTBI population (28, 29, 31, 37, 42, 44). Out of these six studies, three studies were conducted by group A (28, 29, 31). The one study that did not find any significant differences between the two populations at this time point, had a very small sample size (*n* = 5) for each group (46).

Out of the 16 studies comparing IL-6 levels, only 35.2% of the studies completely and 35.2% of the studies partially controlled for the confounding inflammatory variables. The remaining studies (29.6%) did not control for any confounding inflammatory variables. Confounding inflammatory variables include factors such as infections, auto-immune diseases, anti-inflammatory drug intake and other conditions that affect cytokine levels.

#### 3.3.2. TNF-α

Our review identified 15 studies comparing circulating TNF-α levels between patients with mTBI and healthy controls (29, 31, 33–37, 39, 41, 42, 44, 46–49) (Supplementary Figure S2). Out of these, four studies (25.6%; *n* = 4/15) showed significantly elevated TNF-α levels, and one found significantly reduced levels in patients with mTBI at a minimum of one time-point, compared to healthy controls (42, 44, 46, 48, 49). Although all of these studies looked at blood cytokines, one looked at CSF and found elevated TNF-alpha associated with mTBI (48).The remaining 10 studies showed no significant differences in the TNF-α levels between cases and controls (29, 31, 33–35, 37, 39, 41, 42, 47).

From the studies comparing TNF-α levels, 60% (*n* = 9) of the studies completely and 20% (*n* = 3) partially controlled for confounding inflammatory variables. The remaining studies did not control for any confounding inflammatory variable. There was a subject overlap to some extent between the two studies conducted by Group A (29, 31) and three studies by Group C (34–36).

#### 3.3.3. IL-10

Ten studies assessed IL-10 levels in the blood following an mTBI (29, 31, 33–37, 41, 42, 44) (Supplementary Figure S3). This review identified that two out of these ten studies (34, 42) found significantly elevated levels; whereas one study found significantly reduced levels (44) in mTBI patients at a minimum of one time-point, compared to healthy controls. The remaining studies found no significant differences between the two populations.

Out of all studies comparing IL-10 levels, 60% (*n* = 6/10) of the studies completely and 30% (*n* = 3/10) partially controlled for confounding inflammatory variables. The one remaining study did not control for any confounding inflammatory variables. There was a subject overlap to some extent between the two studies conducted by Group A (29, 31) and three by Group C (34–36).

#### 3.3.4. IL-1β

A total of eight studies compared IL-1β levels in blood between mTBI patients and healthy controls (29, 31–33, 41, 42, 47, 50) (Supplementary Figure S4). Three out of eight studies showed significantly elevated IL-1β levels in patients with mTBI compared to healthy controls at a minimum of one time point (31–33, 42, 50). On the other hand, one study showed significantly reduced IL-1β levels in patients with mTBI compared to healthy controls (41). The remaining four studies, however, found no significant IL-1β level differences in blood between the cases and controls (29, 47).

Out of the eight studies comparing IL-1β levels, 37.5% (*n* = 3) of the studies completely and 62.5% (*n* = 5) partially controlled for the confounding inflammatory conditions. There was a subject overlap to some extent between the two studies conducted by Group A (29, 31) and the two by Group B (32, 33).

#### 3.3.5. IL-8

Eight of the included studies assessed IL-8 levels in blood following an mTBI (33, 39, 41, 42, 44, 49–51) (Supplementary Figure S5). Three of the included studies showed significantly elevated IL-8 levels in patients with mTBI, compared to healthy controls (42, 49, 51). Furthermore, two studies showed a significant reduction in IL-8 levels in the mTBI population when compared to healthy controls (41, 44). The remaining studies showed no significant differences in IL-8 levels between the cases and controls.

Out of the eight studies measuring IL-8 levels in blood, 62.5% (*n* = 5) completely and 37.5% (*n* = 3) partially controlled for the confounding inflammatory conditions.

#### 3.3.6. IFN-γ

Six of the included studies assessed IFN-γ levels in blood following an mTBI (29, 33, 39, 42, 44, 49) (Supplementary Figure S6). Out of these, three studies showed significantly elevated IFN-γ levels in patients with mTBI, when compared to healthy controls at a minimum of one time point (42, 44, 49); whereas the remaining three studies showed no significant differences between the two populations (29, 33, 39).

Within 24 hrs, two out of three studies measuring blood IFN-γ levels showed elevated levels in mTBI population, but one study did not find any significant differences between the two populations during this period (29, 42, 44).

Out of the six studies comparing IFN-γ levels, 67% (*n* = 4) of the studies completely and 33% (*n* = 2) partially controlled for the confounding inflammatory variables.

#### 3.3.7. IL-1RA

Blood IL-1RA levels were assessed in five of the included studies (28–31, 49) (Supplementary Figure S7). Out of these, three studies showed significantly elevated IL-1RA levels in patients with mTBI when compared to healthy controls at less than 24 hrs (28, 29, 31). The two remaining studies showed no such differences at any time point (30, 49).

Of the five studies assessing IL-1RA levels, 20% (*n* = 1) of the studies completely controlled for the confounding inflammatory variables whereas 80% (*n* = 4) partially controlled for them. It should be noted that there was a subject overlap to some extent between the four studies conducted by Group A (28–31).

#### 3.3.8. IL-4

Circulating IL-4 levels were assessed in four of the identified studies (33, 41, 42, 44) (Supplementary Figure S8). Out of these, one study showed significantly elevated IL-4 levels in patients with mTBI, compared to healthy controls at a minimum of one time point (42). The remaining three studies, however, did not report any significant differences (33, 41, 44).

Out of the four studies comparing IL-4 levels, 75% (*n* = 3) of the studies completely and 25% (*n* = 1) of them partially controlled for the confounding inflammatory conditions.

#### 3.3.9. MCP-1/CCL2

Circulating MCP-1/CCL2 levels were assessed in eight of the identified studies (32, 33, 38, 39, 41, 47, 49, 52) (Supplementary Figure S9). Out of these, four studies reported significantly elevated MCP-1/CCL2 levels in the mTBI population when compared to healthy controls (32, 33, 38, 49); whereas one study reported a significant reduction in MCP-1/CCL2 levels in the mTBI population (52). The remaining studies showed no significant differences in MCP-1/CCL2 levels between the two populations.

Within 1 week, four out of six studies measuring MCP-1/CCL2 showed elevated levels in the mTBI population (32, 33, 38, 49) but the remaining two studies found no significant differences between the two populations at this time point (39, 52).

Out of the eight studies comparing MCP-1/CCL2 levels, 37.5% (*n* = 3) completely and 50% (*n* = 4) partially controlled for the confounding inflammatory conditions. The remaining studies did not control for any confounding inflammatory variables. There was a subject overlap to some extent between the two studies conducted by Group D (38, 39) and the two by Group B (32, 33).

### 3.4. mTBI vs. healthy controls: meta-analysis of blood inflammatory cytokines

Eleven studies (12 cohorts) involving 987 participants with mTBI were utilized to conduct the meta-analyses for IL-6, TNF-α, IL-1β, and MCP-1/CCL2 levels in the blood. The results show significantly higher circulating levels of IL-6, IL-1β, and MCP-1/CCL2 in the mTBI population in the acute stages (within 1 week), compared to healthy controls. No differences were observed for any inflammatory cytokine in the chronic stages.

#### 3.4.1. IL-6

**Figure 3:**
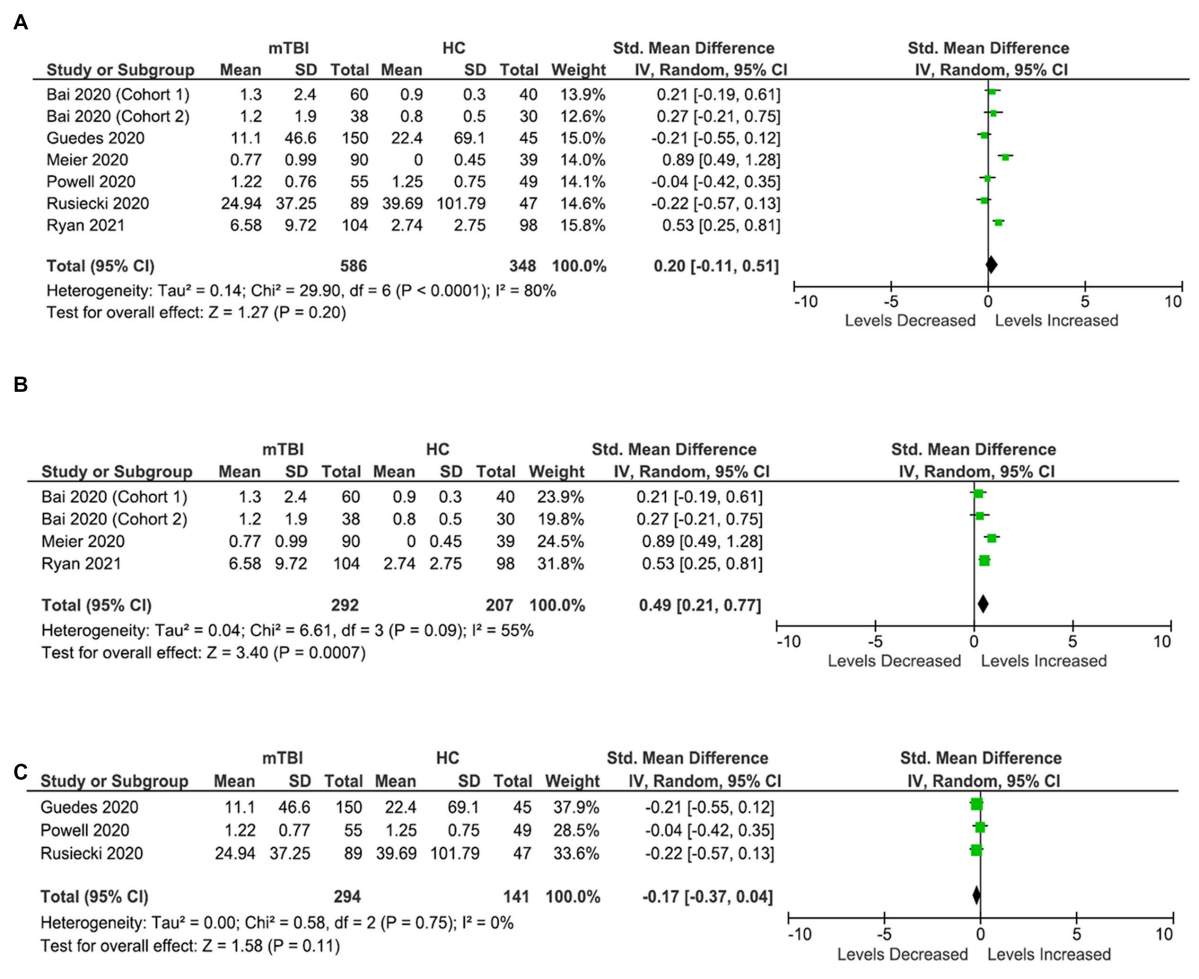
IL-6 meta-analysis. **(A)** All studies. **(B)** Acute IL-6 meta-analysis. **(C)** Chronic IL-6 meta-analysis.

Six studies (seven cohorts) were included in the IL-6 analysis, involving 586 participants with mTBI and 348 healthy controls (Figure 3). The analysis shows no significant differences in the levels of IL-6 in blood between the two populations (SMD: 0.2 [95% CI: −0.11, 0.51] pg/mL, *p* = 0.20, *I*^2^ = 80%) (Figure 3A). The large heterogeneity is partly due to inconsistent results from the included studies due to differences in the timings of assessment, the fraction of blood specimen analyzed, techniques of biomarker assessment, and inflammatory confounding variables.

Further sub-analysis based on timing, i.e., acute and chronic stages showed significantly elevated circulating IL-6 levels in mTBI population compared to healthy population in the acute stages (less than 7 days) (SMD: 0.49 [0.21, 0.77] pg/mL, *p* = 0.0007, *I*^2^ = 55%) (Figure 3B). However, no significant differences were observed between the two populations (SMD: −0.17 [95% CI: −0.37 to 0.04] pg./mL, *p* = 0.11) in the chronic stages (more than 6 months) (Figure 3C).

#### 3.4.2. TNF-α

**Figure 4:**
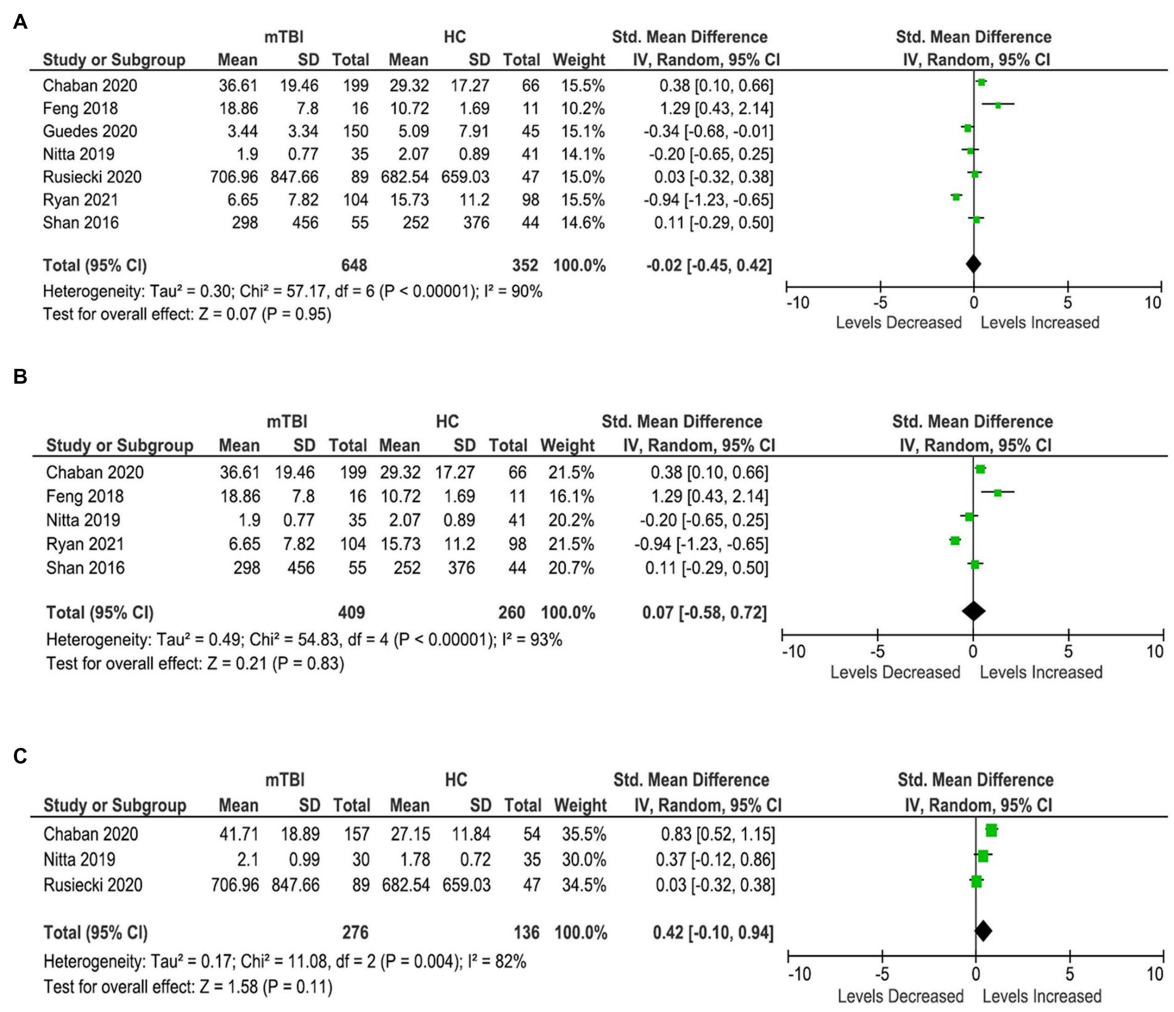
TNF-α meta-analysis. **(A)** All studies. **(B)** Acute TNF-α meta-analysis. **(C)** Chronic TNF-α meta-analysis.

Seven studies were included in the TNF-α meta-analysis, involving 648 participants with mTBI and 352 healthy controls (Figure 4). The analysis shows no significant differences in the levels of TNF-α in the blood between the cases and controls (SMD: −0.02 [95% CI: −0.45, 0.42] pg/mL, *p* = 0.95, *I*^2^ = 90%) (Figure 4A).

Further sub-analyses also showed no differences between the two populations at both acute and chronic stages (Figures 4B,C), and heterogeneity remained high.

#### 3.4.3. IL-1β

**Figure 5:**
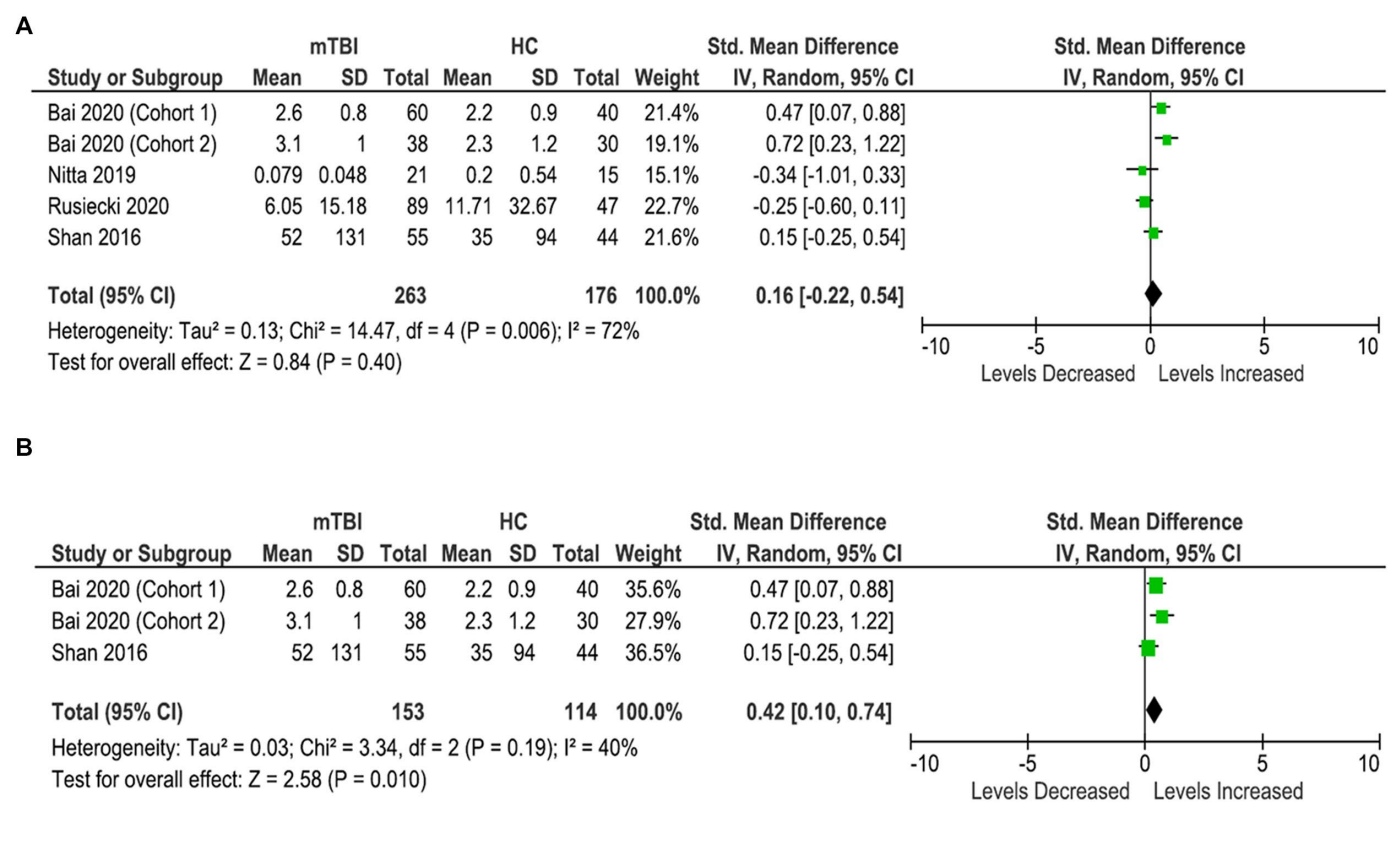
IL-1β meta-analysis. **(A)** All studies. **(B)** Acute IL-1β meta-analysis.

Four studies (five cohorts) were included in the IL-1β analysis, involving 263 participants with mTBI and 176 healthy controls (Figure 5). The analysis showed no significant difference in the levels of IL-1β in the blood between the cases and controls (SMD: 0.16 [95% CI: −0.22, 0.54] pg/mL) (*p* = 0.40, *I*^2^ = 72%) (Figure 5A).

Further sub-analyses showed significantly elevated blood IL-1β levels in the mTBI population in the acute stages (< 7 days) and brought down the heterogeneity (SMD: 0.42 [95% CI: 0.10, 0.74] pg/mL) (*p* = 0.01, I^2^ = 40%) (Figure 5B). A meta-analysis on chronic levels could not be conducted due to an insufficient number of studies.

#### 3.4.4. MCP-1/CCL2

**Figure 6:**
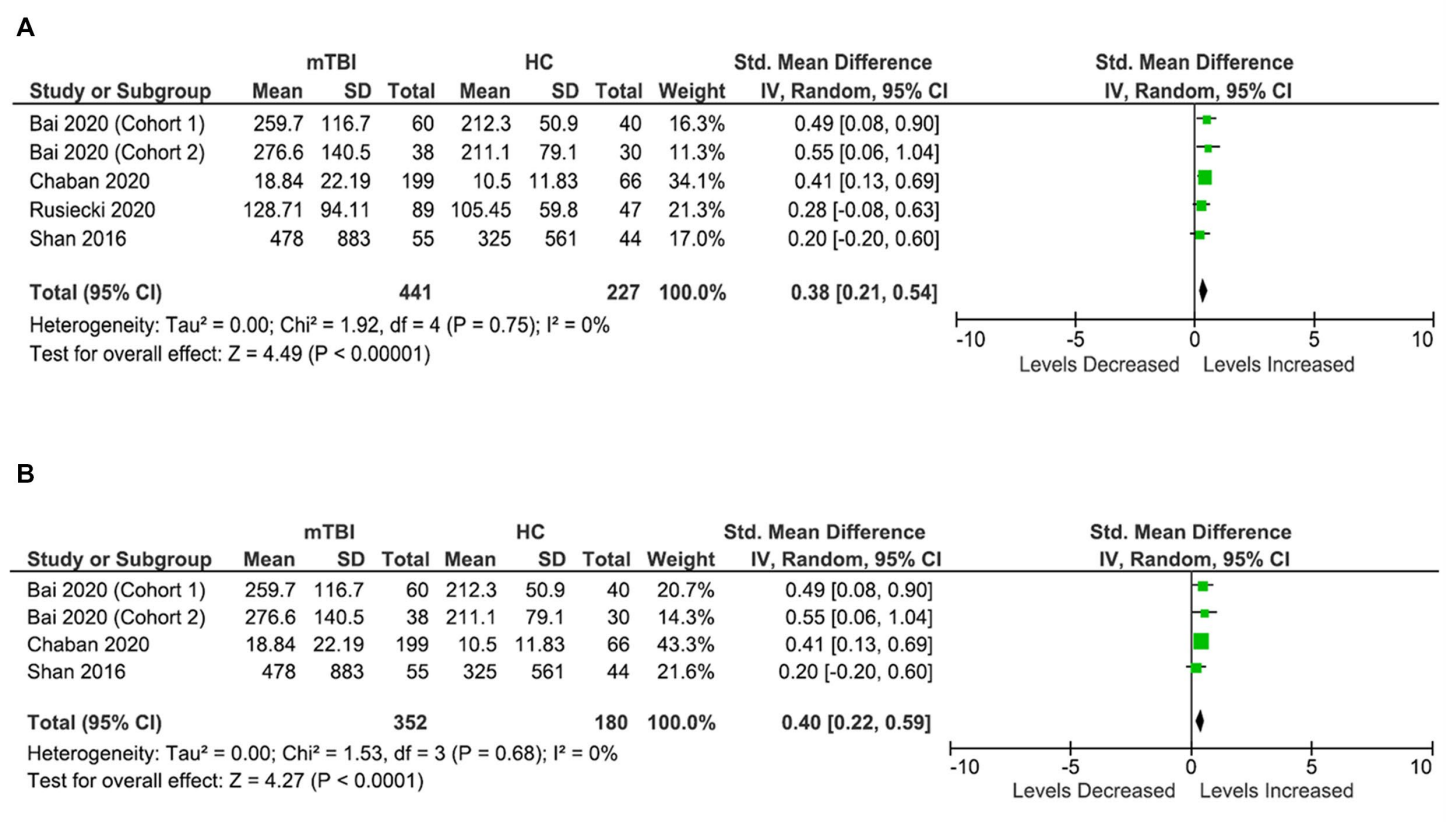
MCP-1/CCL2 meta-analysis. **(A)** All studies. **(B)** Acute MCP-1/CCL2 meta-analysis.

Four studies (five cohorts) were included in MCP-1/CCL2 analysis, involving 441 patients with mTBI and 227 healthy control subjects (Figure 6). Patients with mTBI had significantly elevated concentrations of MCP-1/CCL2 (SMD: 0.38 [95% CI: 0.21, 0.54] pg/mL) (*p* = 0.00001, *I*^2^ = 0%) (Figure 6A) in the blood compared to healthy controls.

Further sub-analysis based on timings demonstrated that blood MCP-1/CCL2 levels are particularly elevated in the acute stages in patients with mTBI compared to healthy controls (within 7 days) (SMD: 0.40 [95% CI: 0.22, 0.59] pg/mL) (*p* = 0.0001, *I*^2^ = 0%) (Figure 6B). Sub-meta-analysis on chronic levels was not possible due to insufficient data.

### 3.5. mTBI vs. trauma controls

Only three studies compared inflammatory cytokine levels in the blood between the patients with mTBI and trauma controls (38, 47, 53).

Shan et al. found no significant differences in the TNF-α, IL-1β, and the chemokines (CXCL1, CXCL8, and MCP-1/CCL2) levels in acute stages (<24 h) between the two groups (47). Vedantam et al. observed significantly elevated IL-2 and IL-6 levels in patients with mTBI in the acute stages (<24 h); however, in the chronic stages only IL-6 levels remained elevated (53). Battista et al. found that athletes with sport-related concussion had higher levels of the chemokines’ monocyte chemoattractant protein-4 (MCP-4) (*p* < 0.001) and macrophage inflammatory protein-1β (MIP-1β) (*p* = 0.001) compared to healthy athletes (within 1 week of injury). At medical clearance, there were no significant biomarker contributions towards the class separation between athletes with SRC vs. healthy athletes (39).

### 3.6. Prognosis

The relationship between inflammatory cytokines in the blood and mTBI prognosis was analyzed in 13 studies (Table 1).

For this analysis, the population was considered to have poor functional outcomes if they had any of the following conditions:

- Persistent symptoms (including emotional/psychological)
- Reduced or lack of return to normal activities (work, school, and sports)
- Abnormal neurocognitive tests/functioning
- Low GOSE scores (<8).

#### 3.6.1. IL-6

Some studies showed that elevated IL-6 levels in the blood at 6 h post-mTBI are significantly associated with the duration of symptoms (*p* = 0.031) (28, 29, 31). On the contrary, Battista et al. showed that there is no significant correlation between IL-6 levels and either symptom burden or days to medical clearance (40).

Guedes et al. found a mild correlation between elevated IL-6 levels in the blood and PTSD in the chronic stages (35).

#### 3.6.2. MCP-1/CCL2

Acutely elevated MCP-1/CCL2 levels in the blood are associated with greater PCS severity and are positively associated with information processing speed at three months post-injury (33). Similarly, acutely elevated MCP-1/CCL2 levels in the blood (within 1 week) are positively correlated with days to recovery in athletes with sport-related mTBI (39). On the other hand, Begum et al. reports that reduced serum MCP-1/CCL2 levels in blood are associated with an increase in the number (r = 0.455, *p* = 0.013) and severity of symptoms (r = −0.378, *p* = 0.043) (52).

#### 3.6.3. TNF-α

Plasma TNF-α levels correlate with persistent PCS and PTSD symptoms (35, 36). Within the mTBI groups, increased circulating TNF-α concentrations is associated with greater PTSD symptoms (*r* = 0.36, *p* = 0.005) (36).

#### 3.6.4. IL-1RA

Acutely elevated circulating IL-1RA levels (within 6 h of mTBI) appear to be significantly associated with greater symptom duration (*p* = 0.03) (28). In addition, there is a significant interaction between prior concussions and levels of IL-1RA on the ImPACT Memory Composite scores (*p* = 0.044) (30). At low levels, athletes with multiple mTBIs show worse memory performance than those without prior mTBIs (*p* = 0.014). Overall, elevated levels are associated with greater symptoms (higher BSI-GSI scores, χ2(1) = 3.98, *p* = 0.046) and worse memory (ImPACT Speed Composite scores, χ2(1) = 5.67, *p* = 0.017) (30).

#### 3.6.5. IL-10

At three months post-mTBI, elevated circulating IL-10 levels are found to be related to PTSD symptoms (B = 0.8, t = 2.60, *p* < 0.01) (34). At the six month mark, elevated plasma IL-10 levels are associated with greater depression scores (*p* = 0.004) and more severe PTSD symptoms (*p* = 0.001) (53).

## 4. Discussion

This study reports significantly higher blood concentrations of IL-6, CCL-2/MCP1, and IL-1β in subjects with mTBI, compared to healthy controls, particularly in the acute stages. While both positive and negative results have been reported for the individual studies, this report strengthens the evidence that mTBI is accompanied by a peripheral inflammatory response (15, 17, 18, 54–56).

Despite extensive knowledge about the protracted recovery and long-term consequences of mTBI, challenges associated with its diagnosis, prognosis, and management remain unresolved. This could be partly attributed to a lack of understanding of mTBI pathophysiology. mTBI appears to be a multifaceted problem, with various biological and non-biological factors at play that determine the clinical outcome (57–59). Neuroinflammation constitutes one of the many secondary pathologies associated with mTBI and represents only a single piece of an intricate puzzle (60). Understanding this neuroinflammation would unravel one of many unknowns of mTBI. To achieve this objective, we have conducted a systematic review and meta-analysis to consolidate and analyze the data on the inflammatory cytokines associated with mTBI. As a result, we are able to identify a few circulating inflammatory cytokines associated with mTBI. The results of the systematic review show significantly elevated levels of IL-6, IFN-γ, IL-1RA (within 24 h), and MCP-1/CCL2 (between 1–7 days) in blood in patients with mTBI, compared to healthy controls. These results are further supported by the results of the meta-analysis which demonstrate significantly elevated blood levels of IL-6, IL-1β, and MCP-1/CCL2 levels in mTBI during the acute stages (within a week). Taken together, these results show a strong association between elevated IL-6, IL-1β, and MCP-1/CCL-2 levels in blood and mTBI during the acute stages. However, due to inherent heterogeneity associated with cytokine-related data, these findings should be interpreted with caution.

IL-6 is a non-specific indicator of inflammation. It is one of the most frequently measured cytokines in mTBI studies. Our review shows that circulating IL-6 levels are consistently higher in patients with mTBI, compared to healthy controls, in the majority of the studies (65%, *n* = 11/17), especially during the acute stages. Interestingly, blood IL-6 levels also seem to be elevated in individuals with mTBI, when compared to those with trauma controls, particularly during the acute phase (53). Apart from IL-6, the peripheral inflammatory cytokine profile associated with mTBI appears to be quite different than the one associated with bodily trauma controls (39). However, due to limited availability of data, no meaningful inferences can be drawn on the circulating inflammatory cytokine level differences between the patients with mTBI and trauma controls without further research. This review also shows that acutely elevated IL-6 levels in blood are consistently associated with poor prognosis, particularly in terms of duration of symptoms (28, 29, 31). However, one study found no significant correlation between IL-6 levels in blood and either symptom burden or days to medical clearance (40). This discrepancy can be attributed to differences in the timings of cytokine level measurements, as Battista et al. (40) measured IL-6 levels in the late acute stages compared to others. Chronically, IL-6 appears to be associated with PTSD (35). Overall, these findings indicate that circulating IL-6, while not highly specific, is a strong indicator of mTBI in early acute stages and could be used to predict clinical outcomes.

MCP-1/CCL2, belongs to the chemokine family of cytokines and is also a non-specific marker of inflammation. This review uncovers a strong association between elevated blood MCP-1/CCL2 levels and mTBI. This association is particularly strong within the first week following an mTBI, as 66% of the studies measuring blood MCP-1/CCL-2 show elevated levels in patients with mTBI, compared to healthy controls. This finding is further supported by the results of the meta-analysis. Beyond 1 week, MCP-1/CCL2 levels remain elevated, extending into the chronic stages; however, the evidence is more robust within 1 week of the mTBI. With regards to prognosis, the evidence is quite conflicting as some studies indicate associations between elevated levels of MCP-1/CCL-2 levels and greater symptom severity, days to recovery, and information processing speed (33, 39). On the other hand, Begum et al. reports that reduced serum MCP-1/CCL2 levels are associated with an increase in the number (r = 0.455, *p* = 0.013) and severity of symptoms (r = −0.378, *p* = 0.043) (52). In addition, due to limited data available, no meaningful inferences can be drawn on MCP-1/CCL-2 level differences between the patients with mTBI and trauma controls without further research. Overall, we can infer that MCP-1/CCL2, just like IL-6, is also a strong indicator of acute mTBI and could be used to predict clinical outcomes.

This meta-analysis also shows significantly elevated IL-1β levels in the acute stages (within a week), compared to healthy controls. TNF-α is the second most common cytokine explored in mTBI studies. This review, however, is unable to detect any significant differences in TNF-α levels between the patients with mTBI and healthy controls. In addition, despite the evidence of elevated IL-1RA, IL-8, and IFN-γ levels in patients with mTBI, particularly within 24 h, we were not able to conduct a meta-analysis due to a limited number of studies.

While this review suggests that TNF-α (35, 36, 61), IL-1RA (28, 30) and IL-10 (34, 53), in addition to IL-6 (29, 31, 35, 36) and MCP-1/CCL2 (33, 39) have the potential to predict the outcome of mTBI, this data is too limited to draw concrete conclusions about these associations.

Neuroinflammation plays both a protective and detrimental role in mTBI (15, 17, 62). While it usually offers neuroprotection early on after an mTBI, persistent neuroinflammation appears to be associated with poorer outcomes (62). As a result, further research is necessary to study the levels of inflammatory cytokines in the chronic stages and their association with persistent symptoms and recovery. Identifying these chronic cytokines may not only be beneficial in monitoring the prognosis of mTBI but may also aid in developing and monitoring targeted treatment strategies for persistent post-concussive symptoms. Although it appears promising, the inherent non-specific nature of these cytokines makes them an unsuitable candidate for the suggested use when employed alone. Recently, many specific markers of neuronal injury, such as UCHL1, GFAP, and S100B have gained much popularity as specific markers of brain injury. Future research may consider utilizing these cytokines in combination with neuronal injury markers to assess prognosis and monitor treatment efficacy, as suggested by others (63). Additionally, future studies may benefit from measuring cytokines and conducting clinical assessment longitudinally at multiple time points to fully understand the relationship between the biomarker recovery trajectory and mTBI recovery trajectory (64).

This study highlights the significant heterogeneity in blood-based inflammatory cytokine data related to mTBI. We acknowledge this limitation and recommend that future studies adopt standardized cytokine analysis methods to minimize data heterogeneity and associated outliers that can result in a 100-fold change across studies. This heterogeneity not only jeopardizes data reliability, accuracy, and reproducibility but also hinders progress in the field. MacDonald et al. recognized and elaborated on these limitations and proposed potential solutions to mitigate them. Incorporating these strategies in future research will help address this issue (63).

### 4.1. Limitations

Our findings must be interpreted with caution.

First, this review shows that there is considerable heterogeneity in the data, leading to difficulties in pooling and analyzing the data to formulate a meaningful conclusion. Heterogeneity was caused by many reasons, some of which include differences in the time elapsed between the initial mTBI and blood sample collection, cytokine analysis technique, blood fraction used for analysis, confounding variable control, mTBI diagnostic criteria, data reporting and functional outcomes measured. Hence, there is a need for a standardized approach in acquiring and reporting data to allow for comparisons.

Secondly, the results of this review show that about 76% of mTBI patients were male. This is because most studies are conducted in the military (27%) and sports populations (42%), which happen to be male-dominant settings. Since females are more at-risk for poor recovery and develop persistent symptoms more frequently compared to males (65–67), we could not assess prognosis accurately based on this data. Future studies, especially those assessing prognosis in mTBI patients, may want to incorporate more female participants in their studies.

Thirdly, the cytokine alterations observed in the mTBI population do not necessarily reflect a pathophysiology associated with head injury alone. There are other variables that should be considered while measuring cytokine levels as they are known to cause considerable fluctuations. These include time of blood collection, sex differences, other injuries (orthopedic, whiplash, muscle strains, etc.) at the time of mTBI, acute and chronic illnesses, co-existing psychiatric conditions, and medication intake amongst others. While we attempted to take some of these limitations into account, the results should still be interpreted with caution due to the factors mentioned above.

Lastly, most studies reported their results using medians or log-transformed means. Although this way of reporting leads to more consistent results, a thorough meta-analysis cannot be conducted using medians as we have found in this review. Future studies should follow a more standardized methodology so that the data reported using means and SD is not as heterogeneous and is more consistent to allow for a more thorough analysis.

### 4.2. Strengths

This systematic review and meta-analysis has several strengths. An exhaustive effort was made to capture the data by searching a variety of databases and acquiring the missing data by directly contacting the authors, extracting data from graphs and tables, or using standardized estimation methods for calculating the mean (SD). Although the latter two strategies may not be accurate, they provide estimates that are closer to the real value and are frequently employed in meta-analyses.

A systematic review with a similar aim was recently published (68). However, our study stands out because it included 15 additional studies. Hence, due to the availability of more data, we were able to conduct a meta-analysis that has not been accomplished before. Visser et al., just like our study, was able to identify IL-6 as a promising biomarker for brain injury (68). It was, however, unable to establish an association between other cytokines (such as MCP-1/CCL2 and IL-1β) and mTBI as we did.

### 4.3. Conclusion

Overall, we found substantial evidence of increased inflammatory cytokine levels in patients with mTBI. The evidence was particularly strong for IL-6, IL-1β, and MCP-1/CCL2. The results of this study were however limited by low study numbers as well as methodological heterogeneity between the studies.

## Author contributions

SM and MR defined the research of interest and were involved in topic selection. SM and OA carried out the literature search and developed Table 1. SM, MM, and OA collected the data. FF, SM, and OA performed the meta-analysis. SM wrote the first draft of the manuscript and prepared all the figures. MM, OA, TG, FF, and MR reviewed the manuscript and made contributions for improvement. All authors helped to revise the paper. All authors contributed to the article and approved the submitted version.

## Funding

This research was supported by McMaster University, Canada.

## Conflict of interest

The authors declare that the research was conducted in the absence of any commercial or financial relationships that could be construed as a potential conflict of interest.

## Publisher’s note

All claims expressed in this article are solely those of the authors and do not necessarily represent those of their affiliated organizations, or those of the publisher, the editors and the reviewers. Any product that may be evaluated in this article, or claim that may be made by its manufacturer, is not guaranteed or endorsed by the publisher.

## Supplementary material

The Supplementary material for this article can be found online at: https://www.frontiersin.org/articles/10.3389/fneur.2023.1123407/full#supplementary-material
