# Supplementary material for: Inflammatory cytokines associated with mild traumatic brain injury and clinical outcomes: a systematic review and meta-analysis
Source: Front Neurol. 2023 May 12;14:1123407. doi: 10.3389/fneur.2023.1123407 (PMC10213278; doi:10.3389/fneur.2023.1123407)
Supplement: Supplementary file 1 [file Table_1.DOCX]

Supplementary Material

**Systematic Cytokines Associated with Mild Traumatic Brain Injury and Clinical Outcome: A Systematic Review and Meta-Analysis**

Shazia Malik ^1^, Omar Alnaji ^2^, Mahnoor Malik ^3^, Teresa Gambale ^4^, Forough Farrokhyar ^5^ *, Michel Rathbone ^4^

^1^ Neurosciences Graduate Program, McMaster University, Hamilton, Canada.

^2^ The Faculty of Life Sciences, McMaster University, Hamilton, Canada.

^3^ Bachelor of Health Sciences Program, McMaster University, Hamilton, Canada.

^4^ Department of Medicine, Division of Neurology, McMaster University, Hamilton, Canada.

^5^ Department of Surgery and Department of Health Research Methods, Evidence, and Impact, McMaster University, Hamilton, Ontario, Canada.

*** Correspondence:**Shazia Malik

Neurosciences Graduate Program, McMaster University,

1280 Main Street West, Hamilton, Ontario, Canada L8S 4L8

Email: [maliks15@mcmaster.ca](mailto:maliks15@mcmaster.ca)

Michel P. Rathbone M.B., Ch.B., Ph.D., FRCP(C)

Department of Medicine, Division of Neurology, McMaster University,

1280 Main Street West, Hamilton, Ontario, Canada L8S 4L8

Email: [mrathbon@mcmaster.ca](mailto:mrathbon@mcmaster.ca)

# Supplementary Figure

**Supplementary Figure 1.** Sampling points for IL-6

**Supplementary Figure 2.** Sampling points for TNF- α.

**Supplementary Figure 3.** Sampling points for IL-10.

**Supplementary Figure 4.** Sampling points for IL-1β.

**Supplementary Figure 5.** Sampling points for IL-8.

**Supplementary Figure 6.** Sampling points for IFN-γ.

**Supplementary Figure 7.** Sampling points for IL-1RA.

**Supplementary Figure 8.** Sampling points for IL-4.

**Supplementary Figure 9.** Sampling points for CCL-2/MCP-1.

.
