# Supplementary figures and images for: Inflammatory cytokines associated with mild traumatic brain injury and clinical outcomes: a systematic review and meta-analysis
Source: Front Neurol. 2023 May 12;14:1123407. doi: 10.3389/fneur.2023.1123407 (PMC10213278; doi:10.3389/fneur.2023.1123407)

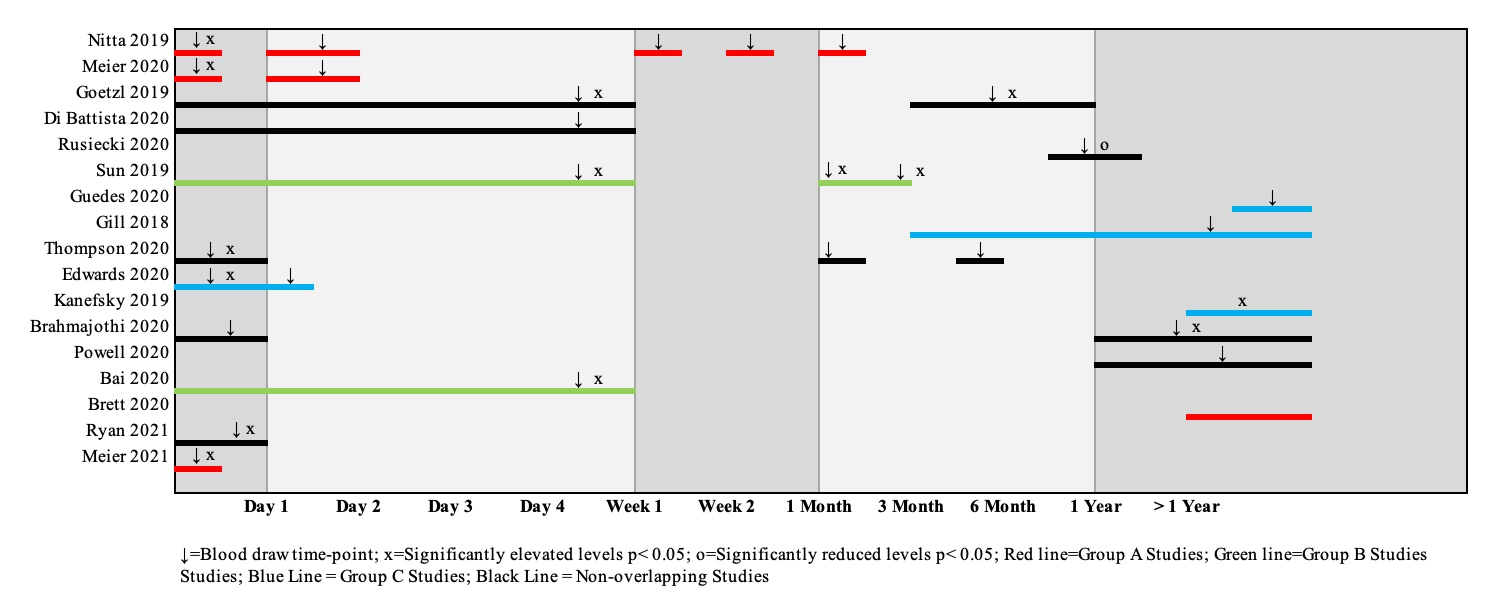

Supplement: Supplementary file 2 [file Image_1.JPEG]

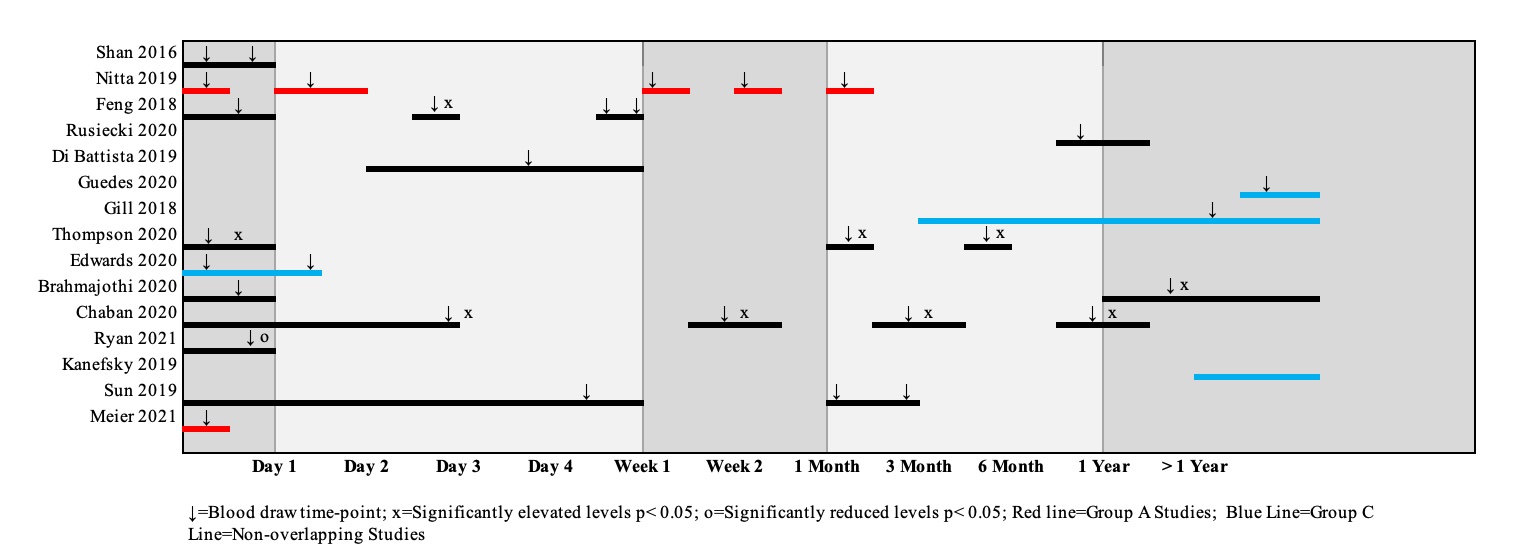

Supplement: Supplementary file 3 [file Image_2.JPEG]

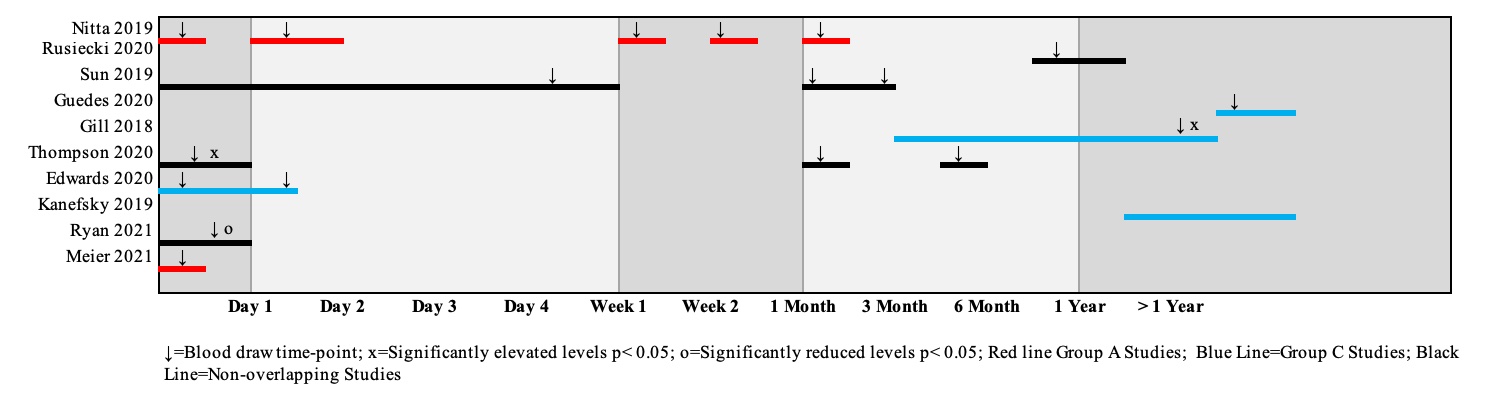

Supplement: Supplementary file 4 [file Image_3.JPEG]

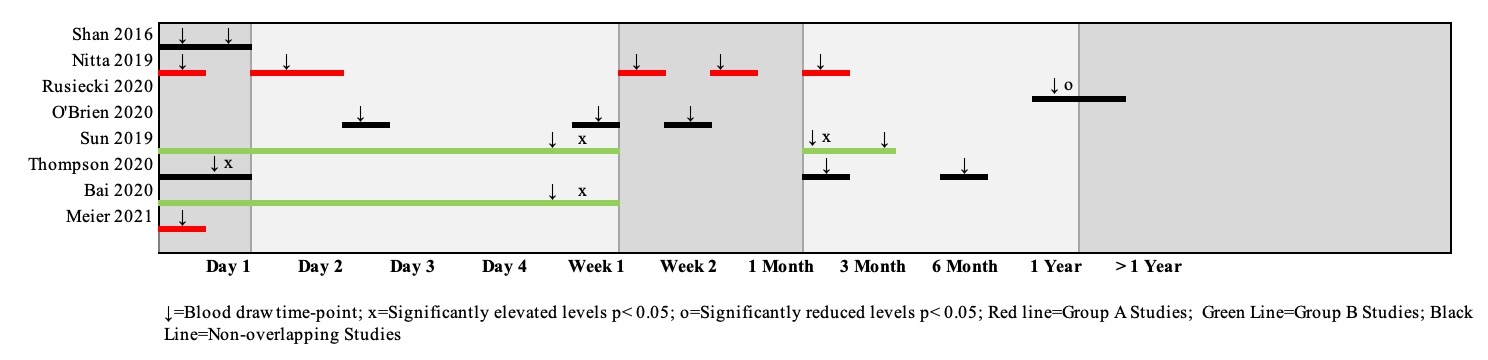

Supplement: Supplementary file 5 [file Image_4.JPEG]

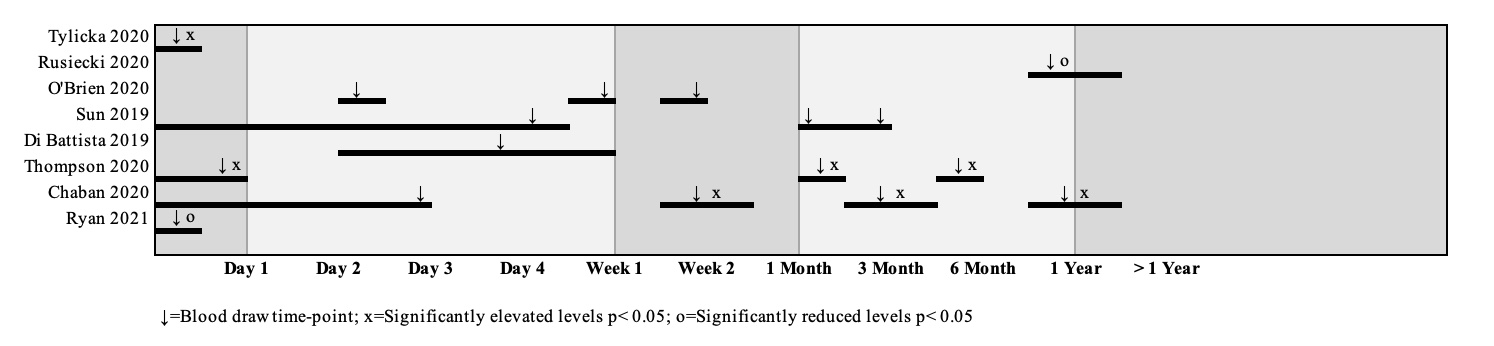

Supplement: Supplementary file 6 [file Image_5.JPEG]

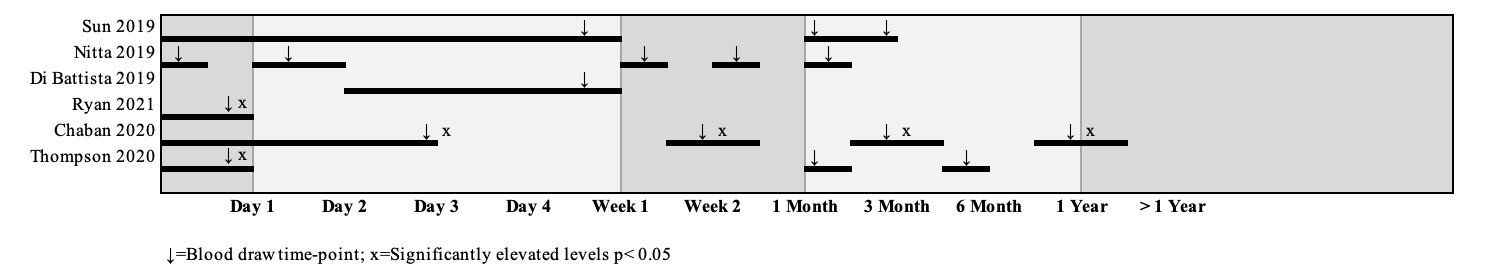

Supplement: Supplementary file 7 [file Image_6.JPEG]

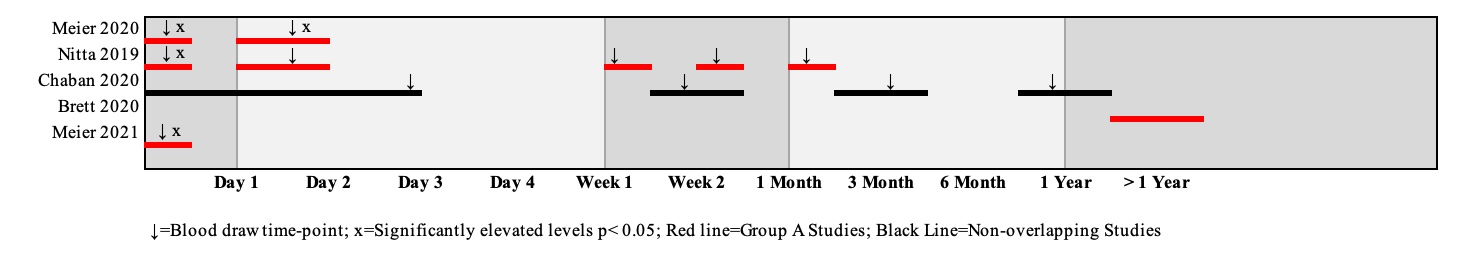

Supplement: Supplementary file 8 [file Image_7.JPEG]

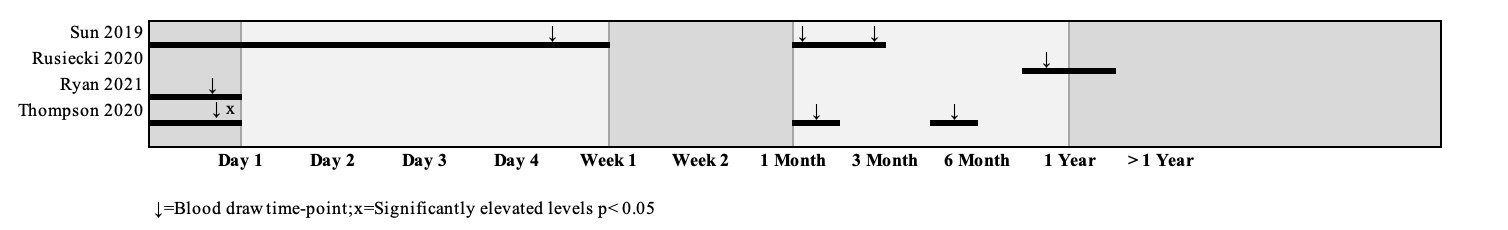

Supplement: Supplementary file 9 [file Image_8.JPEG]

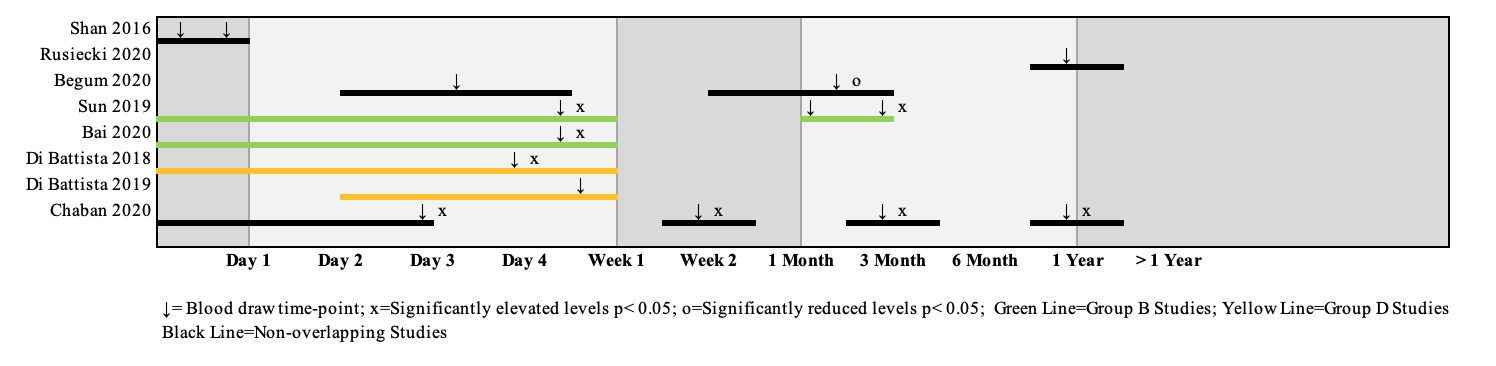

Supplement: Supplementary file 10 [file Image_9.JPEG]
